# Ddx4^+^ Oogonial Stem Cells in Postmenopausal Women’s Ovaries: A Controversial, Undefined Role

**DOI:** 10.3390/cells8070650

**Published:** 2019-06-28

**Authors:** Erica Silvestris, Paola Cafforio, Claudia Felici, Gennaro Cormio, Stella D’Oronzo

**Affiliations:** 1 Gynecologic Oncology Unit, I.R.C.C.S-Giovanni Paolo II Cancer Institute, 70124 Bari, Italy; 2 Department of Biomedical Sciences and Human Oncology, Section of Internal Medicine and Clinical Oncology, University of Bari Aldo Moro, 70124 Bari, Italy; 3 I.R.C.C.S-Giovanni Paolo II Cancer Institute, 70124 Bari, Italy

**Keywords:** Ddx4, oogonial stem cells, infertility, ovarian failure, menopause

## Abstract

Recent studies support the existence of oogonial stem cells (OSCs) in the ovarian cortex of different mammals, including women.These cells are characterized by small size, membrane expression of DEAD(Asp-Glu-Ala-Asp)-box polypeptide-4 (Ddx4), and stemness properties (such as self-renewal and clonal expansion) as well as the ability to differentiate in vitro into oocyte-like cells. However, the discovery of OSCs contrasts with the popular theory that there is a numerically defined oocyte pool for female fertility which undergoes exhaustion with menopause. Indeed, in the ovarian cortex of postmenopausal women OSCs have been detected that possess both viability and capability to differentiate into oocytes, which is similar to those observed in younger patients. The pathophysiological role of this cell population in aged women is still debated since OSCs, under appropriate stimuli, differentiate into somatic cells, and the occurrence of Ddx4^+^ cells in ovarian tumor samples also suggests their potential involvement in carcinogenesis. Although further investigation into these observations is needed to clarify OSC function in ovary physiology, clinical investigators and researchers studying female infertility are presently focusing on OSCs as a novel opportunity to restore ovarian reserve in both young women undergoing early ovarian failure and cancer survivors experiencing iatrogenic menopause.

## 1. Introduction

The theoretical physiology of mammalian fertility has long supported the belief that a fixed pool of oocytes, including approximately 100 progenitors during the time of puberty in women, is committed to providing the mature oocytes that span the duration of female fertility, and that this ovarian reserve undergoes a progressive decrease with aging until complete exhaustion occurs at menopause [1]. Along with the recent evolution of major studies in the field of stemness and regenerative medicine, basic science and clinical researchers investigated the supposed existence of oogonial stem cells (OSCs) that, for their staminal properties (including self-renewal, clonal expansion, and predictable differentiation into oocyte-like cells), would offer an interesting application in redressing ovarian failure in infertile women [2].

Observations of OSCs by independent groups of investigators provided evidence of the existence of OSCs within the murine ovarian cortex [3,4,5]. Important to OSC detection and isolation was the proof that OSCs express the DEAD (Asp-Glu-Ala-Asp)-box polypeptide-4 (Ddx4) molecule, a primordial oogonial marker encoded by the relative gene as an ATP-dependent RNA helicase belonging to the DEAD-box protein family. This protein is also expressed by spermatogonial progenitors and is involved in several cellular processes regulating RNA secondary structure, including the initiation of translation as well as both nuclear and mitochondrial splicing, and the assembly of ribosomes and spliceosomes [6,7].

The orthologue of Ddx4 was originally discovered in *Drosophila* as vasa [6,8]. A vasa gene homologue was then demonstrated in mice as mouse vasa homologue (MVH), and its protein product was described as occurring in early gonadal primordial germ cells until the post meiotic stage in both female and male animals [9]. In humans, the Ddx4 gene, a homologue of MVH, is specifically expressed in the germ cell lineage of both spermatogonia and oogonia, and the relative protein is suspected to play a pivotal role in germ cell development and division in testis and ovary, respectively [10,11].

Although Ddx4 is a component of germ cell granules and was originally described in the cytoplasm of these cells, recent studies have suggested that the C-terminal domain of the protein is expressed on the cell surface of a small subset of human ovarian cells located within the cortex. In fact, in addition to their detection, by using specific antibodies to this portion of the molecule, it is even possible to isolate these cells by immunomagnetic procedures to enrich the Ddx4^+^ cell subset [10]. Such characteristics have led to the consideration of these cells as OSCs, and further studies have convincingly confirmed their stemness properties relative to their abilityto differentiate into oocyte-like cells in vitro [12]. However, thedemonstrated occurrence of OSCs in the ovaries of postmenopausal women raises several concerns regarding their functional role in all phases of a woman’s reproductive life as well as whether or not these cells permanently rest in physiologically dormant conditions [13]. Additionally, there remains the question of the desirability of translating these cells in programs investigating regenerative medicine.

The primary utilization of OSCs is putatively directed at reconstituting the ovarian repertoire in women with ovarian insufficiency because of the ability of OSCs to differentiate into mature, competent, and functional oocytes both in vitro and in vivo, as detected in mice [3]. Thus, in addition to their potential application in the early exhaustion of ovarian reserve, OSCs could be used for fertility reconstitution after gonadotoxic cancer treatments as well as to reverse reproductive senescence induced by age. Moreover, given their biological properties of secreting female hormones, OSCs could also offer a novel approach to control postmenopausal complications of hormonal imbalance in aged women, including cardiovascular diseases, osteoporosis, cognitive decline, and depression [13].

Beyond these potential applications of OSCs, there is speculation as to the pathophysiological role of these cells, in particular in the ovarian cortex of postmenopausal women. The expression of Ddx4 on these cells confirms their staminal condition; this potentially may be related to the activation of unknown molecular mechanisms, thereby driving pathological events in aged ovaries such as cancer, rather than merely guaranteeing an ovarian reserve with aging. 

Here, we review the current literature on OSCs, focusing on their debated pathophysiological role in post-menopausal age, while describing preliminary data by our group in support of the high plasticity and pluripotency of these cells, even in menopause.

## 2. Major Aspects of Ddx4^+^ OSCs

The traditional theory regarding a fixed ovarian reserve during the female mammal reproductive lifetime prevailed until 2004, when Tilly and colleagues provided the first evidence of OSC existence in the postnatal mammalian ovary [14].

By enumerating the follicles in ovarian sections, Tilly et al. [14] revealed a discrepancy between the rate of follicle depletion and length of the mouse’s reproductive life, and thus hypothesized the existence of an alternative source of oocytes. Immunohistochemistry of both young and adult mouse ovarian sections revealed the presence of MVH^+^ cells expressing synaptonemal complex protein (SYCP3), a meiotic cell marker, in the ovarian surface epithelium (OSE). These cells were shown to incorporate bromodeoxyuridine into their DNA, thereby supporting the ability to sustain follicle renewal in relation to mitotic activity. Also, they found healthy maturing follicles in the ovaries of mice with ovarian failure after sterilization by intensive busulfan treatment. Finally, once ovarian fragments of wild-type adult mice were implanted in the ovaries of transgenic mice expressing the reporter gene for green fluorescence protein (GFP), the authors observed the occurrence of GFP-negative oocytes surrounded by GFP-positive granulosa cells, suggesting that the transgenic OSCs migrated into the graft and developed new follicles in adult mice. For their ability to restore folliculogenesis during postnatal life, these cells were thus considered adult OSCs in mammals [14].

Following their original description of OSCs in the ovarian cortex, Tilly et al. [15] later demonstrated the presence of an OSC reservoir in the bone marrow (BM) of adult mice. In fact, after detecting cells expressing germ cell markers such as octamer-binding transcription factor-4 (OCT-4), MVH, deleted in azoospermia-like (DAZL), STELLA, and FRAGILISin the BM of adult female mice, they transplanted the BM into adult females pre-sterilized with cyclophosphamide and busulfan, and recorded the generation of oocyte-containing follicles [15].

However, both isolation and culture of OSCs remained elusive until 2009, when Zou and co-workers [3] successfully isolated female OSCs from the ovaries of both 5-day-old and adult mice. The putative OSCs were obtained by two-step enzymatic digestion of murine ovarian cortex, followed by immunomagnetic isolation based on extracellular MVH expression. In long-term cultures, these cells formed compact clusters and exhibited normal karyotype as well as high telomerase activity. Moreover, these cells expressed germline markers such as OCT-4, MVH, DAZL, FRAGILIS, and STELLA, while oocyte-specific markers, including folliculogenesis-specific basic helix-loop-helix (FIGLA), sex determining region Y-Box-2 (SOX-2), and zona pellucida-3 (ZP3), were undetectable (Table 1). To further support their staminal ontogeny, when MVH^+^ cells were infected with the MSCV-PGK-GFP viral vector and injected into the ovaries of infertile mice, these cells underwent spontaneous differentiation into oocytes from which viable GFP-positive offspring were generated [3].

In a subsequent study, Tilly and co-workers [10] purified mitotically active OSCs from the ovarian cortex of fertile women by sorting Ddx4^+^ cells that appeared very small (4 μm diameter) and expressed primordial germline markers. However, once established in vitro, these cells underwent differentiation to mature oocytes, with a larger diameter of up to 50 μm, and expressed terminal differentiation markers such as growth differentiation factor-9 (GDF-9), ZP glycoproteins, newborn ovary (NOBOX), and both meiosis markers Y-box (YBX)-2 protein and SYCP3. The authors extended these data in vivo by injecting GFP-transduced Ddx4^+^ cells into human ovarian cortical biopsies and observed the formation of follicles containing GFP-positive oocytes 2 weeks after xenotransplantation into NOD/SCID mice. They concluded that the ovaries of fertile women, in a manner similar to those of the adult mouse, harbor mitotically active germ cells capable of generating oocytes both in vitro and in vivo [10].

In agreement with these findings, other authors have described in the OSE of different mammals two separate populations of OSCs, namely very small embryonic-like stem cells (VSELs) with a 2–4 µm diameter and slightly bigger (5–8 µm) committed progenitors [16,17]. The former was considered to be putative pluripotent stem cells, due to their capability to undergo asymmetric division and self-renewal, whereas the latter were presumed to originate from VSEL differentiation.

Despite these results, a few controversial observations were also raised (Supplementary Table S1). Zhang and co-workers [18] used a genetic approach by developing transgenic mice (Rosa26rbw/^+^;Ddx4-Cre) to follow in vivo both proliferation and differentiation of Ddx4^+^cells. In this mouse model, Ddx4 promoter was found to drive the Cre recombinase expression in germline Ddx4^+^cells with a final effect of inducing recombination at a rainbow cassette composed of four open reading frames (ORFs) coding different fluorescent proteins. Thus, Ddx4 expressing cells were distinguished from somatic cells, namely Ddx4-negative cells, and in postnatal mouse ovaries the former were found to be mitotically inactive and did not contribute to oocyte formation during the de novo folliculogenesis. Moreover, in cultures of ovarian cells from these transgenic mice, only Ddx4-negative cells formed colonies with a morphology similar to OSCs. However, reverse transcriptase-polymerase chain reaction (RT-PCR) analysis demonstrated that these cells were not germline for their lack of germ markers such as OCT-4, STELLA, or Ddx4, or the pluripotent stem cell marker SOX-2. These results refreshed the traditional view that neo-oogenesis does not occur in mammals after birth and that no mitotically active Ddx4^+^germline progenitors exist in adult mouse ovaries [18].

Subsequently, Niikura and co-workers [4] reported in their study the presence of putative OSCs in aged mice that underwent folliculogenesis only when transplanted back into a young mouse ovary, thus implying that the surrounding ovarian environment could have a role in sustaining woman’s reproductive function. The authors thereforeconcluded that the principle in which menopause does not allow neo-oogenesis was incorrect and that the two phenomena may coexist [4].

Beyond animal models, in 2013 Stimpfel and colleagues [2] extended these observations in human studies and confirmed the existence of a pluripotent stem cell population in adult ovaries. In their work, small fragments of ovarian cortex from 18 adult women, after enzymatic digestion, were cultured and unexpectedly formed colonies in 17 samples, thus generating small round cells with diameters of up to 4 μm. The cells variably expressed pluripotency markers such as OCT-4, NANOG, and STELLA, in addition to Ddx4 as a germinal lineage marker, and M-CAM/CD146, Thy-1/CD90, and STRO-1 as multipotency markers. These findings support the evidence that adult human ovaries also housed Ddx4^+^ cells with a high degree of plasticity.

In a subsequent study, Clarkson and co-workers [19] reported the detection, isolation, and analysis of a high number of Ddx4^+^ cells from adult human ovarian cortex samples, retrieved by mechanical detachment and a fluorescence-activated cell sorting (FACS) procedure. They isolated several Ddx4^+^ cell subsets separated in relation to the expression of distinct Ddx4 transcripts and levels of progenitor cell markers such as aldehyde dehydrogenase 1 (ALDH1) that were able to develop into oocyte-like structures [19].

**Figure 1:**
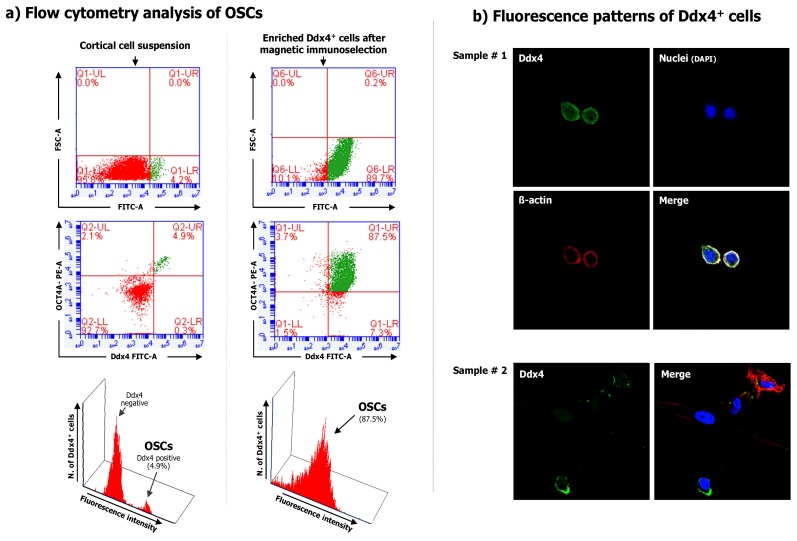
Ddx4 expression on the cell population purified from the ovarian cortex.(**a**) Flow cytometry for Ddx4 expression measured in both total cortical cell suspension (left) and after Ddx4^+^ cell selection emphasized the small population extent in the cortex (4.9%) and its subsequent enrichment (87.5%). (**b**) Immunofluorescence for Ddx4 expression in two samples of purified Ddx4^+^ cells by confocal microscopy. The fluorescence patterns confirm the membrane localization of Ddx4 (FITC; green), while the cell integrity was assessed by both actin (phalloidin; red), and nuclei (DAPI; blue) staining.

We have also recently shown that Ddx4^+^ cells can be detected and purified not only from fertile women but even from those of postmenopausal age [12]. These cells were obtained from ovarian cortex fragments by enzymatic digestion and anti-Ddx4-based immunomagnetic sorting, and confirmed in their phenotype by flow cytometry and fluorescence microscopy (Figure 1).

**Figure 2:**
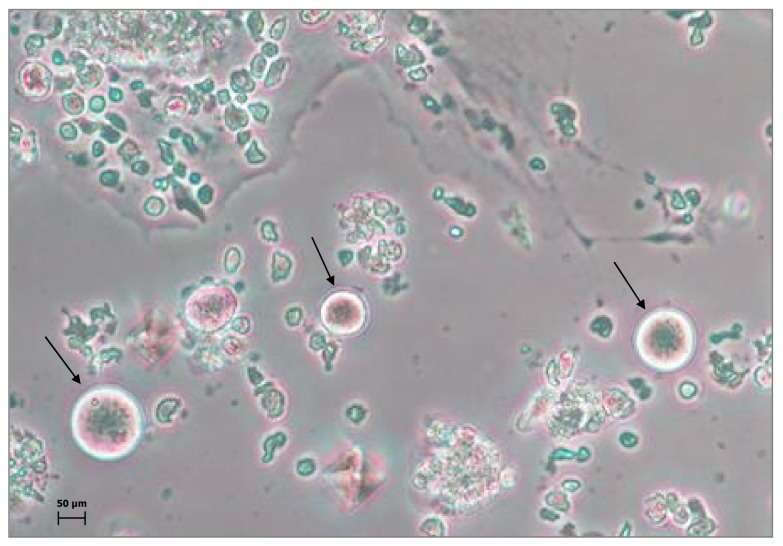
Ddx4^+^ oogonial stem cells (OSCs). Representative image depicts the oocyte-like differentiation of OSCs (black arrows) after 21 days of culture in the presence of fibroblasts feeder layer. Although present in variable size during their differentiation, the largest cells (diameter of approximately 80–90 µm) acquired oocyte-like features, with prominent nuclei and perinuclear accumulation of organelles. By contrast, smaller cells were considered immature OSCs and might represent the oogonial stem cell reserve.

Further phenotype analysis showed that the oogonial markers stage-specific embryonic antigen (SSEA)-4 and FRAGILIS were present on Ddx4^+^ cells to a lesser extent in postmenopausal than in premenopausal women [12]. However, once in culture, several of these cells from both groups differentiated in large cells, with typical oocyte-like morphology, and a diameter of up to 80–90 μm (Figure 2). Furthermore, gene expression analysis confirmed that these large cells expressed variable levels of GDF-9 and SYCP3 mRNA, which were suggestive of terminal oocyte differentiation, while the primordial germ cell marker STELLA was undetectable. Finally, the single signals obtained on X and 5 chromosomes from fluorescence in situ hybridization (FISH) experiments indicated that mature oocyte-like cells were haploid. These results, in combination with the presence of both GDF-9 and SYCP3 mRNAs, suggested the meiotic state of these cells [12].

However, several concerns still remain as to the biologic properties of these cells in adult human ovaries. Both size and quantitation in the cortex are not uniformly assessed by different authors. While Virant-Klun and co-workers defined as mature oocytes cultured cells with a diameter of 35–90 μm [16], we also quantified the cell populations in cultures from non-menopausal and menopausal women [12]. In particular, we observed an inverse correlation between the patient age and the number of isolated Ddx4^+^ cells per cm^3^ of ovarian cortex, but the efficiency of differentiation into mature oocytes did not differ significantly between non-menopausal and menopausal women (*p* = 0.22) [12].

In contrast with our observations, Stimpfel and co-workers [2] observed no significant differences in both the number and colony-forming capability of SSEA4^+^ stem cells derived from the ovarian cortex of women in pre-, peri-, and postmenopausal age, with the exception of a 73-year-old patient whose ovarian cell culture did not generate colonies [2].

Overwhelmingly, the physiopathological role and putative evolutionary meaning of these cells in postmenopausal women have yet to be defined and, although the differentiation of Ddx4^+^ OSCs into oocytes has been repeatedly and well proven in vitro, no data are presently available as to the significance and fate of these cells in vivo, under physiological conditions. It has been demonstrated that OSCs express follicle-stimulating hormone (FSH) receptors and are sensitive to FSH [20], thus suggesting that high serum levels of this gonadotropin naturally occurring in menopause may theoretically contribute to their functional unknown activities or promote deregulated functions as their abnormal proliferation, which may even support cancer development or direct carcinogenesis. 

## 3. Ddx4^+^ Cells and Ovarian Cancer

Although Ddx4 is prevalently expressed in germ cells as a fertility regulator, growing evidence in different organisms describes the involvement of this molecule in several processes of somatic cells and, in some cases, in contributing to tumorigenesis [21]. In this regard, the abnormal expression of Ddx4 in the somatic cells of *Drosophila* has been directly related to similar alterations in brain tumors, supporting the hypothesis that germline molecular traits are involved and contribute to carcinogenesis [22].

In fact, Ddx4 is defined as a translational regulator of specific mRNAs in the germ lineage and participates in the generation of Piwi-interacting RNAs (piRNAs) that protect animal genomes against transposons and are essential for fertility [23,24]. Moreover, Ddx4 expression in somatic cells is tightly regulated and, in several instances, is transient during normal development, while exerting a primary role in the mitotic regulation of embryonic cells and tissue regeneration [25].

Several investigators have demonstrated the occurrence of Ddx4^+^ cells in epithelial ovarian cancer (EOC) samples as well as in ovarian tumor cell lines [26,27,28]. In particular, Hashimoto [26] found that Ddx4 was expressed in 21 out of 75 EOC samples in inclusion cysts with tubal or intestinal metaplasia, considered to be the original site of EOC, and this correlated with both old age of patients and highly malignant serous histology. In addition, by inducing the overexpression of Ddx4 in SKOV-3 ovarian cancer cells, they demonstrated that a number of proteins were up- or down-regulated, and, in particular, that the 14-3-3σ proteinwas down-regulated at the post-transcriptional level. This molecule is a cell cycle regulator induced after DNA damage with a p53-dependent mechanism and is described to play a role in G2 checkpoint, by sequestering the mitotic initiation complex Cdk1-Cyclin B1 in the cytoplasm, with consequent G2 arrest and repair of damaged DNA. For this reason, the down-regulation of 14-3-3σ protein results in the accumulation of chromosomal aberrations that is a cancer hallmark. The authors thus suggested that Ddx4 is potentially involved in EOC progression, and that it is a tumorigenesis marker in ovarian cancer [26].

Moreover, by analyzing 59 samples of different ovarian tumors, Kim and co-workers [27] found that Ddx4 co-localized with CD133, a specific marker of ovarian cancer stem cells [28,29]; interestingly, they also found that CD133 expression was significantly increased in stage IV patients, while both markers were significantly over-expressed in tumors from the oldest patients. This finding reinforced the hypothesis that Ddx4 expression confers the stem signature to ovarian cancer development.

A similar cell population with the stemness phenotype was also found in borderline ovarian cancer [30]. In this tumor, the Ddx4^+^ cells appeared small and round (5 μm diameter) and expressed the pluripotency-related molecule SOX-2 as well as the primordial germ cell marker STELLA along with Ddx4. Once cultured in vitro, these cells proliferated and generated tumor-like structures. Since Ddx4 acts as positive translational regulator of proteins involved in the cell cycle, these results support the hypothesis that Ddx4^+^ cells in EOC represent the cancer stem cell component capable of proliferating and differentiating under specific stimuli. On the other hand, tumor and germ cells share several common features such as high telomerase activity, high proliferation rate, and migration potential, suggesting that the molecular pathways driving carcinogenesis are similarly involved in germ cell development [30].

A stem cell niche prone to malignant transformation was identified in the ovarian hilum region, at the junction between OSE, mesothelium, and tubal epithelium. Although Ddx4 was not investigated in these studies, the niche-forming cells expressed stem or progenitor cell markers such as ALDH1, leucine-rich repeat-containing G-protein coupled receptor-5 (LGR-5), lymphoid enhancer binding factor-1 (LEF-1), CD133, and cytokeratin-6B (CK-6B), and were able to generate tumor spheres in vitro. Their tumorigenic potential was confirmed by silencing TP53 and Retinoblastoma-1 (RB1) as oncosuppressor genes that are frequently mutated in ovarian cancer. In fact, the loss of both proteins induced a significant increase in the proliferation as well as development of high-grade serous adenocarcinoma in xenograft mouse models [31].

**Figure 3:**
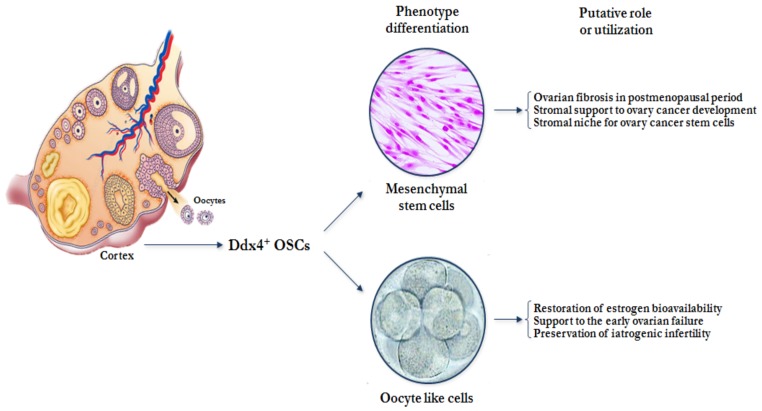
Hypothetical fate of Ddx4^+^ OSCs in post-menopausal women. Under the influence of different hormonal and micro environmental factors related to age or concurrent diseases, Ddx4^+^ OSCs might differentiate into mesenchymal stem cells and/or oocyte-like cells. In postmenopausal women, their mesenchymal differentiation could theoretically support ovarian fibrosis or stromal contribution to cancer development, whereas their oocyte-like induced differentiation could be utilized to restore ovarian hormonal failure or fertility.

Based on these findings, the fate of small Ddx4^+^ cells detected in OSE is still debated, but it is a common opinion that these cells, under different stimuli, are not only capable of promoting neo-oogenesis but also participate in epithelium repair after ovulation and ovarian cancer development (Figure 3) [32].

**Figure 4:**
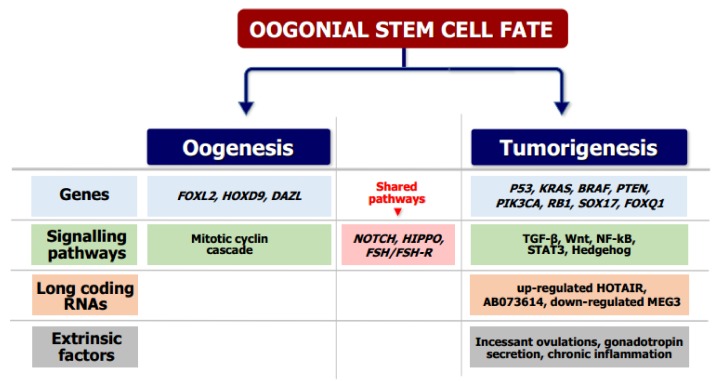
Molecular signals involved in OSC differentiation. OSCs are able to undergo either neo-oogenesis or ovarian tumorigenesis depending on both different stimuli and activated pathways that in some cases are also shared. The molecular signals shown include well-established mechanisms and putative pathways, as reported in the literature [20,25,30,33].

It is well known in ovarian cancer pathogenesis that the cancerous transformation is primed by intrinsic and extrinsic factors, including the multiple activation of signaling pathways that generate the accumulation of gene mutations as well as incessant ovulation during fertile life and the related chronic inflammatory state of OSE. On the other hand, particularly in the postmenopausal age in which the incidence of the ovarian cancer is significantly higher, the chronic hormone stimulation provided by gonadotropins, such as FSH, may drive the acquisitionof molecular derangements of Ddx4^+^ cells that contribute to carcinogenesis [33] (Figure 4).

## 4. Differentiation of Postmenopausal Ddx4^+^ OSCs to Somatic Cells

Recently, increased interest by several investigators has focused on the capability of pluripotent stem cells detectable in human adult ovaries to differentiate in vitro to somatic cells after proper stimuli [2,17,34].

By scraping OSE samples from 21 postmenopausal women without ovarian cancer, Virant-Klun and co-workers [34] purified a subset including a minor number of cells that were interpreted as “putative stem cells”. These cells were of small dimension (up to 4 µm diameter) and round, and were expressing SSEA-4 as well as other stem cell markers, such as C-KIT, NANOG, OCT-4, and SOX-2 [34]. In particular, NANOG, OCT-4, and SOX-2 are considered as master regulators of self-renewal and pluripotency, especially during embryo development, and synergistically modulate their own expression [35]. Virant-Klun et al., also observed that cultures in serum-enriched medium resulted in the development of embryoid body-like structures, together with different types of somatic cells, including neuron-like, myoblast-like, and epithelial cells with progressive loss of stemness markers [34].

A few years later, other authors [17] performed similar scraping of OSE samples from different mammals, including postmenopausal women, and obtained two separate populations of putative stem cells. The former included small-sized cells (1–3 µm) expressing nuclear OCT-4 and surface SSEA-4 molecules, whilst the latter comprised bigger cells (4–7 µm) with very weak SSEA-4 and cytoplasmic OCT-4 expression. Based on these differences, the authors argued that smaller cells were pluripotent stem cells, possibly undergoing asymmetric division to generate committed cells, and this hypothesis was supported by their differentiation in vitro into embryoid body-like structures and neuron-like cells [17].

Hence, these data proved the presence of pluripotent stem cells within the OSE of postmenopausal women, but the existence of similar cell populations in the adult ovarian cortex was described later by Stimpfel and co-workers [2,36]. In particular, following two-step enzymatic degradation with collagenase and hyaluronidase, ovarian cortex samples from peri- and postmenopausal patients without ovarian cancer were cultured and analyzed for the expression of pluripotency and germinal markers, including Ddx4, which were detected in a small cell fraction. These cells were then cultured in separate differentiation media, and somatic cells showing adipocyte-, osteoblast-, neuronal-, and pancreatic-like characters were obtained [2]. This providedproof of the concept of both pluripotency and plasticity of Ddx4^+^ OSCs residing in the adult ovarian cortex.

Also, Stimpfel et al. have recently shownthat the above-mentioned stem cell fraction, namely Ddx4^+^ cells, incorporates mesenchymal stem cells (MSC) expressing CD105, CD44, CD90, CD146, and CD73 as typical markers, and are capable of differentiating into cells belonging to all germ layers, under appropriate stimuli [36]. However, the acquisition of a mesenchymal molecular pattern supports, once again, the somatic differentiation of Ddx4^+^OSCs, while ontogeny-derived MSCs were not investigated in Ddx4 expression in order to speculate their putative germinal origin.

**Figure 5:**
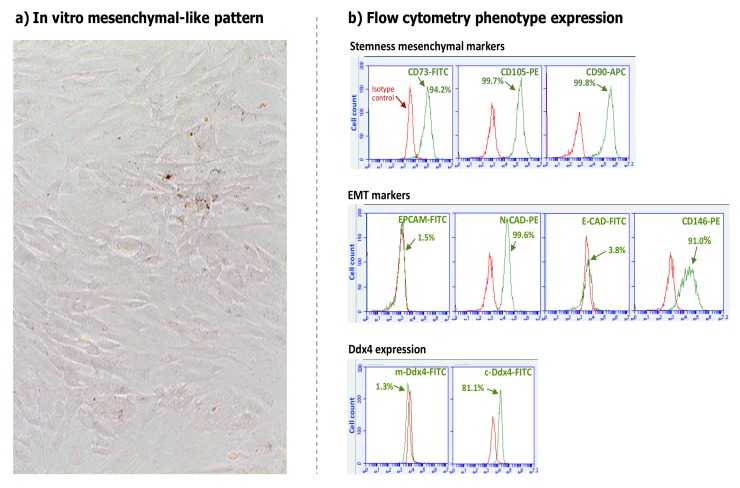
In vitro mesenchymal differentiation and phenotype analysis of cultured Ddx4^+^ cells. (**a**) Light microscope image of 15-day cultured Ddx4^+^ cells in FSH and EGF-supplemented medium showing morphological variation from round to “spindle-like” adherent cells, with a typical aspect of fibroblasts or mesenchymal stem cells. (**b**) Flow cytometry analysis of cultured cells showed a mesenchymal phenotype with typical stemness markers (up), also including the epithelial-to-mesenchymal transition (EMT) molecules as N-cadherin (N-CAD) and CD146, whereas no epithelial markers as EPCAM and E-cadherin, were detected (middle). Based on their differentiation to the mesenchymal phenotype, OSCs lost Ddx4 membrane expression while the molecule was detectable in cytoplasm (down). Red histograms represent isotype controls used in flow cytometry assays.

We have recently explored this aspect in Ddx4^+^ OSCs and found interesting information concerning the putative fate of these cells in ovaries during the postmenopausal stage of women. By applying a previously described protocol [12], we isolated small round Ddx4^+^ OSCs from the ovarian cortex of post-menopausal women undergoing oophorectomy for non-malignant diseases. In order to investigate their capability to acquire a “mesenchymal-like” phenotype, we exposed these cells to both FSH and epidermal growth factor (EGF) that are well known to promote the epithelial-to-mesenchymal transition (EMT) in different cell models [37,38,39,40,41]. Thus, adherent “spindle-like” cells appeared after 48 h of culture and developed a monolayer within about 15 days (Figure 5a). Interestingly, these cells expressed MSC markers (CD90, CD105, N-cadherin, CD146, and CD73) and cytoplasmic Ddx4, while this marker was progressively lost on the cell surface (Figure 5b).

Such a mesenchymal phenotype transition of Ddx4^+^ OSCs under FSH endocrine stimulation may perhaps resemble the hormonal condition typical of the menopausal stage in women in which ovary fibrosis may be dependent on high FSH serum levels.

Further experiments are needed to better characterize the somatic properties of these cells, although the data presently available support the germinal origin of ovarian MSCs and the high plasticity of Ddx4^+^ cells, whose differentiation is possible even in postmenopausal women under the influence of both hormonal and micro environmental factors.

## 5. Potential Application of Ddx4^+^ OSCs in Pre- and Postmenopausal Women

Once the existence of Ddx4^+^ OSCs in the human ovarian cortex and their germ lineage differentiation in vitro after recruitmenthas been definitively determined, it is thus conceivable to consider their future potential applications in regenerative medicine.

During the reproductive period of women until menopause, ovarian neo-oogenesis induced by OSCs may contribute not only to the development of novel techniques to efficiently restore female fertility in several conditions of ovarian failure, such as early ovary exhaustion, but would also enable fertility preservation for oncologic patients undergoing chemotherapy or other gonadotoxic cancer treatments [13]. Despite innovative anti-cancer treatments with small molecules, targeted therapy, and immunotherapy, chemotherapy protocols that are largely adopted resultin a marked reduction of the follicular reserve of fertile women, with a consequent decrease in estradiol levels and an increase in the production of FSH from the pituitary gland, defining a typical endocrine profile of menopause [42].

Particularly in ovarian insufficiency, the recruitment of OSCs and their culture in vitro to generate mature oocytes, followed by fertilization and subsequent implantation in the uterus, would bean attractive alternative to the oocytes obtained from ovulations induced in vivo by repeated hormonal stimulation. This procedure is largely utilized by clinicians in medical centers enrolled in the treatment of infertility in women [43] and, although consequences of chronic oestrogenic stimulation are debated, it cannot be excluded that the persistent hyper-oestrogenism could also increase the risk of hormone-dependent malignancies, such as breast and gynaecologic tumors [44,45]. This aspect is specifically relevant in programs of fertility restoration in premenopausal patients with those tumors, in whom parallel oestrogen-sensitive cancer cell clones derived from the primary tumor undergo molecular derangements of proliferative intracellular signaling, resulting in carcinogenesis [46].

To avoid repeated hormonal stimulations in oncologic premenopausal women, ovary cortex cryopreservation and transplantation to restore a woman’s fertility is currently adopted only in selected institutions and is encumbered by the high risk that transplanted autologous oocytes may not result in natural fertilization, while patients still require, even at a lower dosage, hormone stimulation [47]. By contrast, once appropriate numbers of OSCs are recovered and differentiated in vitro, these cells might be cryopreserved until fertilization, and subsequently implanted in the uterus without the necessity of hormonal treatments. This approach for the restoration of female fertility through autologous OSCs in cancer survivors, as a hormone-free procedure, is safe and may be proposed as an alternative approach for iatrogenic infertility care.

In contrast with the putatively intensive utilization of OSCs in reversing ovarian failure in premenopausal women, the presumptive use of this model of regenerative medicine in the postmenopausal time period appears limited, if contextualized to reproduction. Reproductive aging is indeed characterized by a progressive decline in fecundity and fertility through reduced ovarian function [48], which also contributes to the development of other health complications such as osteoporosis, cardiovascular diseases, cancer, and urinary disorders [49]. Until now, the concept of rejuvenating the ovarian environment to counteract menopause-related disorders by hormone replacement has been described in several studies, principally through ovarian tissue transplantation [50]. In this context, translating OSC technologies into clinical applications may provide a promising innovative strategy for correcting the hormonal imbalance typically associated with ovarian failure, with the advantage of possibly utilizing heterologous OSCs without the need for autologous tissue for each transplantation. However, this would require additional supportive evidence in order to overcome several ethical concerns associated with receiving the implant of germ cells, even for purposes unrelated to reproduction.

In general, the in vitro oocyte recruitment from OSCs provides innovative approaches for the treatment of both primitive and secondary infertility. The main advantage in restoring the fertility of cancer patients by OSCs lays in the opportunity to avoid intensive hormonal stimulation that could increase the risk of metachronous or delayed hormone-dependent carcinogenesis. On the other hand, replacement of oocytes in the ovarian cortex of postmenopausal women may be useful in regulating the hormone imbalance typically encountered in aging and probably may be efficient in restraining the onset of pathological conditions associated with age-related hypo-oestrogenism in postmenopausal women.

## 6. Conclusions

Recent advances in the field of regenerative medicine have focused on the application of OSCs, which, because of their ability to differentiate into oocyte-like cells in vitro, are considered a promising therapeutic approach to infertility and menopause. Indeed, the evidence of OSC occurrence in the ovarian cortex of non menopausal and menopausal women, and the ability of these cells to differentiate to form mature oocytes in vitro, strongly emphasize their putative use in the treatment of ovarian insufficiency.

However, based on their stemness, occurrence in postmenopausal women, and hormone sensitivity, particularly to FSH, it remains a possibility that Ddx4^+^ cells are involved in several biological processes of somatic cells and that they may also contribute to carcinogenesis in ovarian aging. Evidence that small Ddx4^+^ cells, cultured with FSH and EGF, acquire the MSC phenotype supports the concept of pluripotency and plasticity typical of stem cells.

In conclusion, beyond the unknown physiologic role of these cells during postmenopausal age in women, their putative utilization in balancing the endocrine derangements related to aging should be considered in future studies aimedat preventing the severity of some conditions typically associated with defective hormonal function in menopause.

## Figures and Tables

**Table 1:** Cell locations of shared and independently expressed molecular markers by OSCs and mature oocytes [10,13].

Marker	Oogonial Stem Cells	Mature Oocytes
**Ddx4**	membrane and cytoplasm	cytoplasm
**SSEA4**	membrane and cytoplasm	cytoplasm
**DAZL**	nucleus and cytoplasm	cytoplasm
**OCT4A**	nucleus	-
**OCT4B**	cytoplasm	cytoplasm
**c-kit**	cytoplasm	cytoplasm
**Fragilis**	membrane and cytoplasm	-
**Stella**	nucleus and cytoplasm	-
**CD133**	membrane	-
**SOX-2**	nucleus	-
**Nanog**	nucleus	-
**Blimp-1**	nucleus	-
**SYCP-3**	-	cytoplasm
**GDF-9**	-	cytoplasm
**ZP proteins**	-	membrane
**NOBOX**	-	nucleus

Acronyms: Ddx4: DEAD-box polypeptide 4; SSEA-4: stage-specific embryonic antigen 4; DAZL: deleted in azospermia-like; OCT-4: octamer-binding transcription factor 4; ZP: zona pellucida; GDF-9: growth differentiation factor 9; SYCP3: synaptonemal complex protein 3; NOBOX: newborn ovary; SOX-2: sex determining region Y-box 2.

## Supplementary Materials

The following are available online at https://www.mdpi.com/2073-4409/8/7/650/s1, Table S1: The debate on either existence (A) or inexistence (B) of OSCs. (A) Studies supporting the existence of the OSCs. (B) Studies denying the existence of the OSCs.
