# Supplementary material for: Ddx4+ Oogonial Stem Cells in Postmenopausal Women’s Ovaries: A Controversial, Undefined Role
Source: Cells. 2019 Jun 28;8(7):650. doi: 10.3390/cells8070650 (PMC6678385; doi:10.3390/cells8070650)
Supplement: Supplementary file 1 [file cells-08-00650-s001.pdf]

Supplementary Table 1. The debate on either existence (A) or inexistence (B) of OSCs. (A) Studies supporting the existence of the OSCs. B) Studies denying the existence of the OSCs.

References	Model	Main findings
Johnson J et al, Nature 2004	Mouse	Description of mitotically active GSCs in the OSE of young and adult mice, expressing MVH and supporting the follicle renewal.
Bukovsky A, Reprod Biol Endocrinol 2004	Human	GSCs from superficial epithelium of adult human ovaries differentiate to oocytes, granulosa, neural, epithelial and mesenchymal cells.
Kerr JB et al, Reproduction 2006	Mouse	Immature germ cells from adult ovary may sustain de novo oogenesis.
Lee HJ et al, J Clin Oncol 2007	Mouse	Bone marrow transplantation rescues long-term fertility by transferring GSCs that replenish oocytes lost in CTX-treated female mice.
Zhang D et al, Reprod Sci 2008	Mouse	Presence in adult mouse ovaries of cell aggregates that express GL and SC markers.
Szotek PP et al, PNAS 2008	Mouse	LRCs from OSE of transgenic adult mouse (H2B–GFP) show stem/progenitor cell hallmarks.
Virant-Klun I et al, Differentiation 2008	Human	Isolation of putative OSCs with germline features from the OSE of postmenopausal women and those with premature ovarian failure.
Zou K et al, Nat Cell Biol 2009	Mouse	Immunomagnetic isolation of proliferative MVH-positive GSCs from postnatal mouse ovaries expressing pluripotency and GL markers. Following transplantation into infertile mice, GSCs produce viable offspring.
Niikura Y et al, Aging 2009	Mouse	In aged mouse ovaries there are GCs with high expression of the STRA8 and DAZL genes that generate oocytes after transplantation into ovaries of juvenile mice.
Virant-Klun I et al, Differentiation 2008	Human	GSCs from OSE of postmenopausal women generate in vitro embryoid-bodies-similar-structure expressing MVH.
Pacchiarotti J et al, Differentiation 2010	Mouse	Identification of Vasa positive GSCs in adult mouse ovary capable of self-renew and differentiation into oocyte-like cells.
Gong SP et al, Fertil Steril 2010	Mouse	Establishment of two colony-forming cell lines from ovarian tissue having embryonic stem cell activity.
Parte S et al, Stem Cell Dev 2011	Human-Rabbit	Detection of VSELs expressing pluripotent markers, as Oct4 and Nanog able to differentiate to oocyte-like cells.
Song SH et al, Stem Cell Dev 2011	Pig	Isolation of OSCs expressing Oct4, Nanog and Sox2,which are able to generate oocyte-like cells in vitro.
White YAR et al, Nat Med 2012	Human	Ddx4-based isolationby FACS of mitotically active OSCs from both mouse and human ovaries, which spontaneously differentiate into oocytes and generate chimeric follicles when transplanted into immunodeficient mice.
Esmaeilian Y et al, Adv Biosci Biotechnol 2012	Mouse	Detection of PSC markers as Oct4, Nanog and Sox2 in pre-puberal and adult mouse ovaries.
Bhartiya D et al, J Ovarian Res 2012	Human	Pluripotent VSELs and OGSCs of adult mice ovariesare regulated by FSH and are responsible for postnatal oogenesis and follicular assembly in reproductive life.
Patel H et al, J Ovarian Res 2013	Sheep	FSH stimulates self-renewal and differentiation of OSCs through alternatively spliced receptors variant FSH-R3.
Stimpfel M et al, Cell Tissue Res 2013	Human	Ovarian cortex of adult womencontains GSCs that express P and GL germinal markers. SSEA-4-positive cells from culture differentiate into different somatic cells of all three germ layers.
Virant-Klun I et al, Biomed Res Int 2013	Human	Purification of a small SSEA-4-positive SCs from adult OSE with potential embryonic-like featuresusing MACS and FACS.
Sriraman K et al, Reprod Sci 2015	Mouse	VSELs in mouse ovaries survive to chemotherapy, are modulated by FSH and differentiate into oocytesexpressing MHV and GDF9.
Ding X et al, Sci Rep 2016	Human	Oocyte differentiating GSCs from fertile women enter meiosis and produce chimeric follicles in adult immunodeficient female mice.
Silvestris E et al, Hum Reprod 2018	Human	Ddx4^{+}-OSCs from NMW and MW differentiate to large haploid OLCs expressing GDF-9 and SYCP3, and enter meiosis.
Clarkson YL et al, Sci Rep 2018	Human	FACS detection of human OSCs by extracellular DDX4 coupled with ALDH1.

References	Model	Main findings
Bristol-Gould SK et al, Dev Biol 2006	Mouse	Hypothesis of a limited production of oocytes during fetal life, that ceases after birth.
Liu Y et al, Dev Biol 2007	Human	Absence of P and M markers in adult ovaries of healthy women.
Byskov AG et al, Hum Reprod 2011	Human	No detection of PP-GSCs markers such as SSEA-4, Oct4 and Nanog in 2-year-old human ovaries.
Zhang H et al, PNAS 2012	Mouse	Ddx4-expressing GL progenitors are mitotically inactive and do not contribute to the formation of oocytes in adult ovary.
Kerr JB et al, Reproduction 2012	Mouse	No restoration of primordial follicle reserve occur after sterilization treatment with DXR or γ-rays.
Yuan J et al, Stem Cells 2013	Rhesus, Monkey	Absence of proliferative cells and neo-oogenesis in adult monkey ovaries.
Lei L &Spradling AC, PNAS 2013	Mouse	Adult mice ovarydoes not contain mitotically activeGSCs neithergeneratesnovel oocytes in vivo.
Zhang H et al, Curr Biol 2014	Mouse	GSCs in the adult mammalian ovary do not give rise to postnatal oogenesis.
Zhang H et al, Nat Med 2015	Mouse, Human	Ddx4-positive OSCs from human and mouse ovaries are not functional GSCs able to regenerate oocytes.
Zarate-Garcia L et al, Sci Rep 2016	Mouse	Cell sorting method based on membrane Ddx4 marker isolates a small population of ovarian cells that are not GCs and do not express GL markers as Ddx4, DPPA3 and DAZL.

Many groups of researchers have investigated OSCs in different species with different separation methods, but only the groups in the highlighted boxes adopted Ddx4 molecules for isolating the OSC population. Acronyms: Germline stem cells (GSCs), Germ line (GL), Stem cell (SC) markers, Label-retaining cells (LRCs), Ovarian surface epithelium (OSE), Mouse vasa homologue (MVH), Germ cells (GCs), Very small embryonic-like stem cells (VSELs), Oogonial stem cells (OSCs), Fluorescence-activated cell sorting (FACS), Pluripotent stem cell (PSC), Pluripotent and Germinal markers (P and GL markers), Automated magnetic-activated cell sorting system (MACS), non-menopausal (NMW), menopausal women (MW), Growth differentiation factor 9 (GDF-9), Synaptonemal complex protein 3 (SYCP3), Aldehyde dehydrogenase (ALDH1).
